# Displacement affinity chromatography of protein phosphatase one (PP1) complexes

**DOI:** 10.1186/1471-2091-9-28

**Published:** 2008-11-10

**Authors:** Greg BG Moorhead, Laura Trinkle-Mulcahy, Mhairi Nimick, Veerle De Wever, David G Campbell, Robert Gourlay, Yun Wah Lam, Angus I Lamond

**Affiliations:** 1 Department of Biological Sciences, University of Calgary, 2500 University Dr. N.W. Calgary, AB T2N 1N4, Canada; 2 Wellcome Trust Biocentre, MSI/WTB Complex, University of Dundee, Dundee, DD1 5EH, UK; 3 MRC Protein Phosphorylation Unit, School of Life Sciences, University of Dundee, Dundee, Scotland DD1 5EH, UK

## Abstract

**Background:**

Protein phosphatase one (PP1) is a ubiquitously expressed, highly conserved protein phosphatase that dephosphorylates target protein serine and threonine residues. PP1 is localized to its site of action by interacting with targeting or regulatory proteins, a majority of which contains a primary docking site referred to as the RVXF/W motif.

**Results:**

We demonstrate that a peptide based on the RVXF/W motif can effectively displace PP1 bound proteins from PP1 retained on the phosphatase affinity matrix microcystin-Sepharose. Subsequent co-immunoprecipitation experiments confirmed that each identified binding protein was either a direct PP1 interactor or was in a complex that contains PP1. Our results have linked PP1 to numerous new nuclear functions and proteins, including Ki-67, Rif-1, topoisomerase IIα, several nuclear helicases, NUP153 and the TRRAP complex.

**Conclusion:**

This modification of the microcystin-Sepharose technique offers an effective means of purifying novel PP1 regulatory subunits and associated proteins and provides a simple method to uncover a link between PP1 and additional cellular processes.

## Background

The phosphorylation of proteins is one of the most prevalent covalent modifications known, affecting essentially every aspect of cellular function [1,2]. The protein kinases and phosphatases responsible are highly conserved across species and, with few exceptions, the kinases belong to one large gene family while the phosphatase complement is more complex and can be divided into three broad groups based on protein sequence, catalytic signature and substrate preference [3-5]. The action of protein phosphatases is tightly controlled with cellular targeting being an important means of regulation. Most phospho-serine and threonine dephosphorylation can be attributed to the PPM family and the more diverse PPP family, which includes PP1, PP2A, PP2B, and PP4 through to PP7. PP1 is thought to not exist as a free catalytic subunit in the cell, but to reside in complexes with a large array of targeting or regulatory subunits that define its function. Numerous PP1 docking proteins have been identified, but they most likely represent only a small fraction of the total number in the cell.

The microcystins are a group of cyclic peptides that bind with remarkable specificity and affinity to the type one, 2A and several recently identified protein phosphatases of the PPP family (e.g. PP4, PP6). Microcystin covalently couples to a conserved cysteine residue of PPP family members through its methyl-dehydroalanine residue [6,7]. Nishiwaki et al [8] first used Microcystin-Sepharose to purify PP2A. We exploited a different synthetic approach whereby the carbon-carbon double bond of methyl-dehydroalanine in microcystin couples the latter to aminoethanethiol, which is then linked to a Sepharose bead. This generates a high affinity binding matrix for the microcystin-sensitive protein phosphatases that does not covalently couple the phosphatase [6]. Microcystin-Sepharose has proved to be a powerful tool to purify these protein phosphatases and their associated regulatory subunits from a variety of tissues and cell types [9-12].

With only a few characterized exceptions, PP1 interacting proteins bind PP1 through their primary docking sequence called the RVXF/W motif [13]. Their molecular interaction with PP1 has been visualized via PP1-peptide and PP1 regulatory subunit structures [14,15]. It has also emerged that additional or secondary interaction sites often play a role in binding PP1 and likely contribute to PP1 isoform specificity recognition, substrate docking and modulation of PP1 activity [13,15-20]. Based on a compilation of demonstrated RVXF/W interaction motifs [21-23], the panning of a random peptide library [24], and mutagenesis and modeling studies [23] we noted preferences for particular amino acids within and adjacent to the RVXF/W motif. This led us to speculate that a PP1 interaction motif peptide, based on this comparison, could be a unique means to specifically disrupt PP1-targetting or regulatory subunit interactions [14,25,26]. Slight variation in the RVXF/W-motif combined with the now recognized additional, secondary PP1 interaction sites provide sufficient interaction specificity which may allow the development of drugs or peptide mimetics to abolish specific PP1 binding protein interactions *in vivo*. The idea of targeting protein-protein interaction domains with drugs has historically not been favored by the pharmaceutical industry yet, due to improved understanding of the underlying molecular mechanisms, it is now a concept that is growing in popularity [27,28]. This idea has been explored with PKA anchoring proteins (AKAPs) where optimal RI and RII subunit binding peptides were derived from parent peptides and used to target PKA *in vivo *and displace it from its normal anchoring site [29]. In addition to *in vivo *targeting, disrupting PP1-regulatory subunit interactions with a peptide would be an effective means to aid in identifying proteins in PP1 complexes and thus uncover new cellular processes regulated by this protein phosphatase.

## Results and Discussion

**Figure 1:**
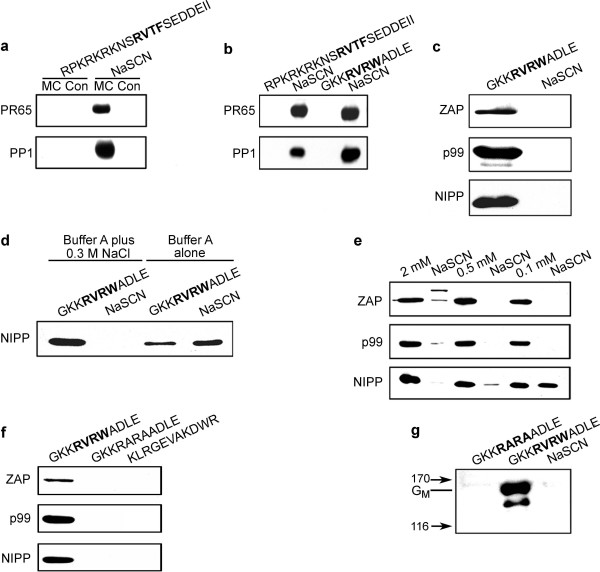
**A peptide based on the PP1 interaction motif RVXF/W displaces PP1 regulatory subunits from the affinity matrix microcystin-Sepharose**. Proteins extracted from isolated rat liver nuclei were incubated with the affinity (MC) or control (Con) matrix (Tris coupled). Proteins were displaced with the RPKRKRKNS**RVTF**SEDDEII peptide (a), while in (b), elution was performed with either RPKRKRKNS**RVTF**SEDDEII or GKK**RVRW**ADLE prior to the 3 M NaSCN elution. The control matrix is only used in the panel (a) experiment while microcystin-Sepharose is used in all others. After concentration, samples were run on 10% SDS-PAGE, blotted to nitrocellulose and membranes were probed with anti-PR65 and PP1 antibodies. In panel (c) the membrane was probed with anti-ZAP, p99 or NIPP1 antibodies [31]. To test for the salt dependence of peptide displacement, columns were eluted with peptide plus or minus NaCl as indicated and samples blotted for NIPP1 (d). To determine optimal peptide concentration for displacement from the column, the protein loaded beads were divided into 3 equal parts and eluted with 0.1, 0.5 and 2 mM peptide (e). To test for the specificity of the peptide displacement, the beads were divided into 3 equal parts and eluted with either GKK**RVRW**ADLE, the GKK**RVRW**ADLE peptide with the key interacting residues changed to A (GKK**RARA**ADLE), or a scrambled version of the GKK**RVRW**ADLE peptide (KLRGEVAKDWR) and blotted for ZAP, p99 and NIPP1. Glycogen particles were isolated from rabbit skeletal muscle and PP1G_M _bound to the microcystin matrix followed by peptide elution first with the GKK**RARA**ADLE peptide, then GKK**RVRW**ADLE, followed by 3 M NaSCN.

We initiated our PP1 peptide displacement study selecting RVXF/W containing peptides from the PP1 targeting subunits NIPP1 [25,30] and ZAP (ZAP3) [31,32]. They were synthesized and tested for their ability to displace PP1 binding proteins from complexes retained on the microcystin matrix. In brief, we isolated rat liver nuclei, extracted proteins and incubated extracts with Microcystin-Sepharose to bind the microcystin-sensitive protein phosphatases [31]. After extensive column washing, we incubated the matrix with the NIPP1 or ZAP peptides (RPKRKRKNS**RVTF**SEDDEII and GKK**RVRW**ADLE, respectively) to selectively displace proteins retained on the matrix through PP1. This implies that PP1 itself and other microcystin-sensitive phosphatase complexes should be retained on the matrix and can subsequently be eluted with the chaotrophic agent sodium thiocyanate (NaSCN), which will also displace other bound proteins [9] including some that may be retained by binding non-specifically to the Sepharose bead. Blotting for PP1 and the PP2A regulatory subunit PR65 show this to be true (Figure 1a and 1b) with no retention of these phosphatase subunits on a control matrix coupled with Tris alone. The NIPP1 peptide (RPKRKRKNS**RVTF**SEDDEII) readily displaced PP1 regulatory subunits (data not shown), yet we chose to continue working with the ZAP peptide (GKK**RVRW**ADLE, later also referred to as RVRW peptide) because it is small, readily soluble and an excellent match to the optimal PP1 binding peptide discovered through panning a random peptide library [24]. Indeed, the ZAP peptide RVRW motif and the additional C-terminal amino acids ADL and N-terminal basic amino acids (KK) were most frequently obtained in the random peptide library screen (Figure 2 in [24]). We thus predict that the ZAP sequence GKK**RVRW**ADLE is the most suitable, high affinity peptide that will compete and displace most other proteins docked to PP1 through variations of the RVXF/W-motif. We acknowledge that even with an 'optimal binding peptide' there are certainly other PP1 interacting proteins on the matrix that may not be displaced because of completely novel PP1 interaction sites and/or additional interaction sites outside of the RVXF/W-motif [20] that could maintain the interaction with PP1 even if the RVXF/W site is displaced. Thus, the PP1 binding partners uncovered here still most likely only represent a sub-population of the total nuclear PP1 binding partners.

**Figure 2:**
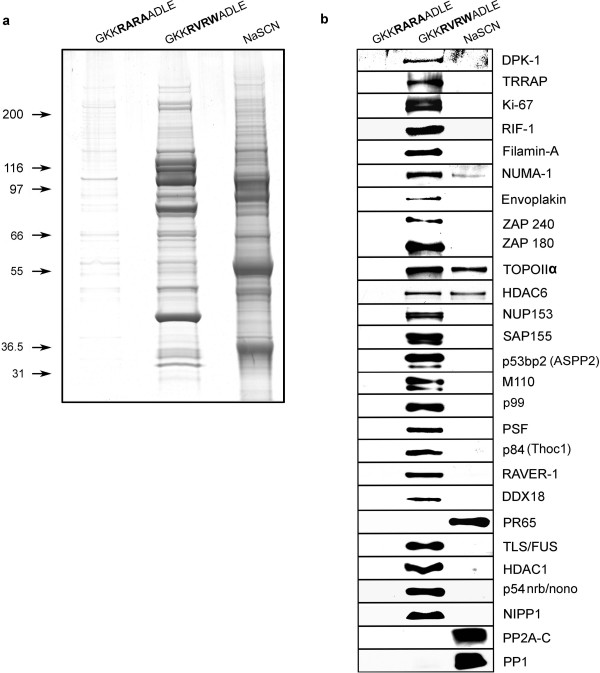
**Displacement affinity chromatography purification of PP1 complexes from HeLa cell nuclei**. (a) A HeLa cell nuclear extract was incubated with microcystin-Sepharose, washed extensively and eluted with the GKK**RARA**ADLE peptide, followed by GKK**RVRW**ADLE peptide and finally with 3 M NaSCN. Each fraction was concentrated to an equal volume, run on 4–12% SDS-PAGE (Invitrogen) and stained with Collodial blue. Individual bands were excised, trypsin digested and identified by mass spectrometry (see Additional files 2 and 4). In (b) the same samples from panel (a) were blotted to a membrane and probed with antibodies to proteins identified by mass spectrometry. Protein names are defined in abbreviations list.

RVRW peptide eluted samples were blotted for the known nuclear PP1 binding proteins ZAP, p99 (PNUTS) and NIPP1 (Figure 1c). This revealed that these proteins were readily displaced from the matrix by the peptide with essentially no further regulatory subunit being eluted with NaSCN. A direct comparison of elution conditions with the GKK**RVRW**ADLE peptide showed that displacement was much more effective in a higher ionic strength buffer (Figure 1d). Having established that the GKK**RVRW**ADLE peptide is an excellent tool to displace PP1 regulatory subunits, we tried several peptide concentrations to optimize displacement from the matrix (Figure 1e). This demonstrated that 0.1 mM peptide was sufficient to displace ZAP and p99, but 0.5 to 2 mM peptide was needed to remove all of the more abundant and perhaps higher affinity binding NIPP1 protein. At the 2 mM peptide concentration we noted that displacement was in fact not as effective for ZAP or p99. In subsequent experiments we eluted by first incubating with 0.5 mM, then 2 mM peptide to ensure all PP1 complex proteins were displaced. To ensure the specificity of the method, we included 2 independent negative controls. Nuclear phosphatases bound to the microcystin matrix were equally divided into 3 parts and eluted with either the GKK**RVRW**ADLE peptide, the GKK**RVRW**ADLE peptide where the key PP1 interacting amino acids (V and W) were replaced by alanine (GKK**RARA**ADLE), or a scrambled ZAP peptide (KLRGEVAKDWR). These fractions were blotted for ZAP, p99 and NIPP1 (Figure 1f) confirming the specificity of the displacement technique.

To extend this method to PP1 complexes outside the nucleus, we isolated glycogen particles from skeletal muscle where the well characterized PP1 regulatory subunit G_M _resides and functions to target PP1 to glycogen [22]. As shown in Figure 1g, the peptide GKK**RVRW**ADLE effectively displaced G_M _from the microcystin-matrix. In addition, prior to the RVRW displacement, we performed an incubation with the GKK**RARA**ADLE peptide and, as predicted, due to the absence of the key V and W residues in the docking motif, this peptide did not displace the G_M _protein from the matrix which supports the idea that the peptide elution is specific for PP1 complexes. All subsequent experiments included the GKK**RARA**ADLE peptide elution and buffer wash step prior to GKK**RVRW**ADLE peptide displacement.

**Figure 3:**
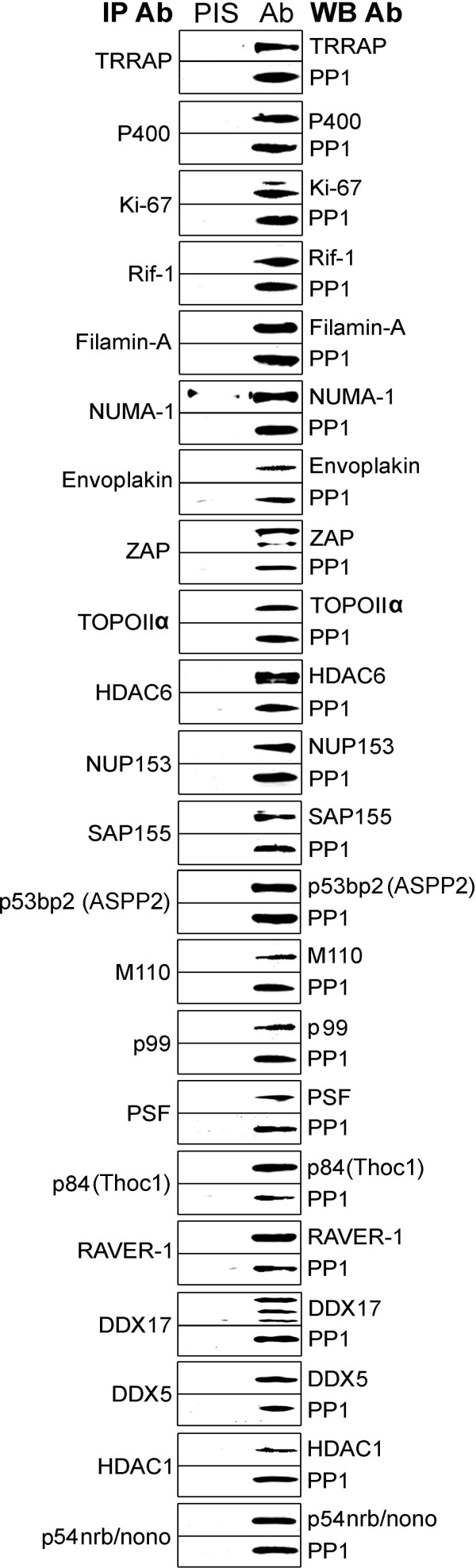
**Co-immunprecipitation of PP1 with HeLa nuclear PP1 binding and complex proteins**. Antibodies were obtained for the proteins shown and immunoprecipitations performed. Immunoprecipitated proteins were run on SDS-PAGE, transferred to a membrane and probed with the same antibodies as indicated plus a pan PP1 antibody to determine if PP1 co-immunoprecipitates. The immunoprecipitating antibody is shown on the left and in all cases a pre-immune serum control was done in parallel (PIS). The blotting antibody it indicated on the right.

Having developed this methodology, we scaled it up to specifically release PP1 interacting proteins from the matrix with the aim of identifying novel, less abundant nuclear PP1 regulatory subunits and/or proteins that reside in PP1 complexes. We predict many more unrecognized PP1 regulatory subunits exist whose identification may have been masked in previous studies when all microcystin-sensitive phosphatases (especially the abundant PP2A complex) and non-specifically bound proteins were released from the matrix using NaSCN alone [31]. Furthermore, peptides may not be detected during mass spectrometry in the presence of more abundant peptides. Using an extract prepared from isolated rat liver nuclei we could show that GKK**RVRW**ADLE peptide displacement gave a banding pattern on SDS-PAGE that was clearly distinct from the subsequent NaSCN elution [Additional file 1]. Individual GKK**RVRW**ADLE eluted bands were excised from the gel and proteins identified by mass spectrometry [Additional file 2]. Many previously characterized and new potential PP1 binding proteins were identified. To validate our findings in different organisms, we then switched our attention to the human HeLa cell line. Here the GKK**RARA**ADLE, GKK**RVRW**ADLE and NaSCN column elutions again show distinct protein patterns (Fig 2a). Mass spectrometry identification of individual bands from the GKK**RVRW**ADLE displacement is shown in Additional file 3 and file 4. For many of the identified proteins we were able to obtain antibodies and blotting of the individual elution steps confirmed that the GKK**RARA**ADLE peptide did not elute any of the known or putative new PP1 interactors, supporting the idea that all of the GKK**RVRW**ADLE released proteins reside in PP1 complexes and that many are direct PP1 binding proteins (Figure 2b). Of note is the abundant presence of putative PP1 docking sites within the sequences of identified proteins (additional files 2 and 4). Although we predict only PP1 binding/complex proteins should elute with docking site peptide, mass spectrometry reveals that some PP1 catalytic and PP2A components did release with GKK**RVRW**ADLE elution, while blotting showed that the vast majority of the total pool of these subunits were retained on the matrix (Figure 2b). This is not surprising given that some complexes likely contain both PP1 and PP2A [33] and other proteins may contain more than one PP1 dock site. The lack of displacement with the GKK**RARA**ADLE peptide, and subsequent elution with GKK**RVRW**ADLE provide compelling evidence that PP1 directly docks or is in these protein complexes. To explore this further we performed immunoprecipitations with antibodies we obtained for many of these proteins and in every instance tested we could demonstrate the interaction with PP1 (Figure 3). These data again support the idea that we specifically eluted PP1 complex proteins from the matrix. One reason for using the RVRW peptide elution was to specifically displace proteins and not release any polypeptides that associate non-specifically. It is known that some proteins bind non-specifically to Sepharose beads and thus we sought to determine if the proteins discovered here were retained on Sepharose alone and eluted with the GKK**RVRW**ADLE peptide. Therefore, as an additional control, we passed a HeLa nuclear extract over Sepharose beads alone, washed as usual, then eluted with the GKK**RARA**ADLE and GKK**RVRW**ADLE peptides and NaSCN, as described for the microcystin-matrix. These samples were then western blotted for a selection of interactors that were previously retained on the microcystin-Sepharose and eluted with the GKK**RVRW**ADLE peptide (Figure 2b). When the entire GKK**RARA**ADLE, GKK**RVRW**ADLE and NaSCN fractions were individually concentrated and run on a single lane and blotted versus 1/175 of the input (which gave a strong western signal) we did weakly detect several of these proteins in the NaSCN elution (as expected), but only noted signals for filamin A and PSF in the GKK**RVRW**ADLE peptide elution (data not shown). Given that this is in comparison to 1/175 of the input, this represents a very minor background displacement and functions to remind us that, as in any exploratory work, additional experiments are needed to confirm true interactors. Interestingly, PSF has previously been defined as a PP1 docking protein [34].

It is notable from the mass spectrometry data that we identified many nuclear processes or complexes not previously linked to PP1. For instance, we found numerous TIP60 (TRRAP) complex proteins [35]. Immunoprecipitation of the TIP60 components TRRAP or p400 (Domino) were able to bring down PP1, confirming that PP1 is a previously unrecognized component of this complex. Putative interacting proteins or complexes identified here will need to be confirmed by co-immunoprecipitation experiments or other methods, and then ultimately all complexes, including the TRRAP complex, will need to be studied to define the role of PP1 binding to that protein or group of proteins.

A comparison of the proteins eluted and identified from rat and human nuclei reveals that most of the proteins are the same (although the number of rat proteins is greater), but several differences are apparent. First, some differences are not surprising given that the HeLa cell line expresses numerous proteins that differ from the rat liver cell proteome. In addition, there are quite a number of proteins in the rat samples that appear in multiple bands excised from the gel, but this only occurs a few times in the HeLa bands. This is likely due to the fact that preparation of the rat nuclei from liver tissue is more time consuming and thus we expect increased proteolysis of proteins and therefore many more hits of the same protein in bands of many masses. Comparing rat liver versus cells grown in culture also means that we started with much more protein from the rat preparation and this certainly contributed to the identification of more lower abundance proteins.

To date several approaches have been used to define the PP1-interactome of the cell, including total (NaSCN) elution from microcystin-Sepharose followed by overlay analysis, SILAC mass spectrometry based PP1 binding partner investigation and most recently an antibody array approach for selected putative targets [11,31,36,37]. Any methodology has its limitations; our work certainly represents a subgroup of the total PP1 targeted protein/complexes of the nucleus for reasons discussed above and because our nuclear extraction procedure likely only released some fraction of the total PP1 complexes. It is clear that many more PP1 binding partners exist than originally thought and it will be the combination of multiple approaches that will ultimately define the plethora of roles for PP1 in the nucleus and other cellular compartments.

## Conclusion

We have developed a method to specifically elute proteins that reside in PP1 complexes or bind directly to PP1 after retention on the affinity matrix microcystin-Sepharose. This approach should be valuable to isolate new PP1 binding proteins and to link PP1 function to as yet unrecognized PP1 regulated cellular processes across a broad range of tissues, cell types and organisms. This technique also lends further credibility to the notion that it could be possible to develop peptides as tools for the specific disruption of protein protein interactions with potential usage for therapeutic purposes.

## Methods

### HeLa nuclear extracts

For HeLa cells, nuclei were isolated and extracted from ~1 × 10^9 ^cells grown in spinner flasks using the method outlined at . Sonicated, extracted nuclei were made to 0.42 M NaCl, mixed end-over-end for 10 min. at 4°C and clarified by centrifugation at 10,000 g for 10 min. This clarified extract was then mixed with the microcystin matrix.

### Affinity displacement chromatography

Microcystin-Sepharose was prepared as described [7,9]. Control matrix was prepared by coupling to Tris instead of aminoethanethiol-microcystin. In a typical nuclear preparation, the livers of 10 rats were homogenized, nuclei isolated and proteins extracted [31]. Nuclear proteins were incubated end over end with 1 mL of microcystin-Sepharose or control matrix for 1 h at 4°C and then washed in a column with 250 mL of buffer A (25 mM Tris-HCl pH 7.5, 0.1 mM EGTA, 1 mM benzamidine, 0.1 mM PMSF, 0.1% β-ME) plus 300 mM NaCl. All peptides were dissolved in buffer A plus 300 mM NaCl and the pH adjusted to 7.5 with 1 N NaOH by spotting on pH paper. The matrix (1 mL) was first incubated with 2 mL 0.5 mM peptide for 30 min. and the eluted protein collected with the addition of 2 mL buffer A plus 300 mM NaCl. The matrix was then incubated with 2 mL 2 mM peptide for 30 min. and the eluted protein collected with the addition of 5 mL buffer A plus 300 mM NaCl. After washing with an additional 30 mL of buffer A plus 300 mM NaCl, the column was eluted with 3 M sodium isothiocyanate in buffer A [9]. The peptides used were RPKRKRKNS**RVTF**SEDDEII (from human NIPP1), GKK**RVRW**ADLE (from human ZAP), GKK**RARA**ADLE (from human ZAP with alanine substitutions for V and W) and KLRGEVAKDWR (scrambled GKK**RVRW**ADLE). The 0.5 and 2 mM peptide elutions were pooled and immediately concentrated in a centriprep 10, then centricon 10 to 40 μL and boiled in 5× SDS-cocktail. The NaSCN elution was dialyzed extensively and concentrated to 40 μL as described for the peptide elutions and boiled in 5× SDS-cocktail. Samples were run on 10% SDS-PAGE or in some cases on 4–12% gradient gels (Invitrogen).

### Isolation of rabbit skeletal muscle glycogen particles and chromatography on MC-Sepharose

Skeletal muscle from the back and hind legs of one rabbit was removed, placed on ice, minced and homogenized with 2.5 L/kg homogenization buffer (2 mM EDTA, 2 mM EGTA, 0.1% B-ME, 1 mM benzamidine, 0.1 mM PMSF) in a blender and centrifuged in a Beckman J6 at 4200 rpm for 30 min. at 4°C. The supernatant was decanted through glass wool in a funnel and the pH of the solution lowered to 6.1 with 1 M acetic acid. After 15 min on ice, glycogen was pelleted by centrifugation at 4200 rpm for 30 min. at 4°C. The glycogen pellet was resuspend in 100 mL of the following buffer (50 mM Tris-HCl pH 7, 2 mM EGTA, 5% V/V glycerol, 4 μg/mL leupeptin, 1 mM benzamidine, 0.1 mM PMSF, and 0.1% β-ME.) and loaded on a 1 mL microcystin-Sepharose column equilibrated with microcystin-Sepharose buffer A. After incubation for 1.5 hour with an end-over-end machine, then washing with microcystin-Sepharose buffer A plus 0.3 M NaCl (200 column volumes or until no protein comes off in a Bradford assay) the matrix was eluted with peptides and NaSCN as described above.

### Mass spectrometry

For protein identification by mass spectrometry bands were excised from colloidal blue stained gels, digested with trypsin and LC-MS/MS was performed as in Ulke-Lemée et al [32]. Peak lists were searched with Mudpit scoring using Mascot version (v2.2). We used the criteria of 2 matching peptides for a positive identification.

### Immunoprecipitations

Antibodies to proteins or an equivalent amount of pre-immune serum purified IgG were coupled to Protein A-Sepharose (GE Healthcare) and incubated with HeLa cell extracts (from 4 × 10 cm plates) end over end for 2 h at 4°C. After sedimentation, IP pellets were washed 2× with PBS, 2× with PBS plus 0.05% NP-40 and 150 mM NaCl and 2 more times in PBS, before boiling in SDS-cocktail. In all cases, antibody and pre-immune serum IPs were incubated with identical amounts of protein, washed in parallel and eluted with an equal volume of cocktail to allow direct comparison on western blots.

Antibodies were kindly provided by individuals or purchased as indicated in brackets. PP1, NIPP1, p99 (PNUTS), SAP155, ZAP and their use are detailed in Tran et al [31]. Other antibodies were PR65 (B. Hemmings), G_M _and M110 (P. Cohen), DPK-1 (SP Lees-Miller), NUP153 (M. Lohka), DDX5 (F. Fuller-Pace), RAVER-1 (B. Jockusch), TOPOIIα (E. Kurz), TRRAP, Envoplakin, HDAC6, PSF (Santa Cruz), Ki-67, p53bp2 [ASPP2], p54nrb/nono (BD Biosciences), Rif-1, p84 [Thoc1], P400 (Abcam), Filamin-A (Chemicon), NUMA-1 (Nova Biologicals), DDX18, DDX17 [DNA helicaseA] (Bethyl Lab), HDAC1 (Cell Signaling), PP2A-C (Transduction Labs).

## Abbreviations

DPK1: DNA-dependent protein kinase; TRRAP: transformation/transcription domain-associated protein; RIF1: Rap1-interacting factor 1; NUMA-1: nuclear mitotic apparatus protein 1; TOPOIIα: topoisomerase IIα; SAP155: spliceosome-associated protein 155; NUP153: nucleoporin 153; M110/MYPT1: myosin targeting subunit 110; p53bp2: p53 binding protein 2; PSF: PTB-associated splicing factor; RAVER1: ribonucleoprotein: PTB-binding 1; PR65: PP2A scaffolding or A subunit; p400/Domino: E1A-binding protein p400; ASPP: apoptosis stimulating proteins of p53; NaSCN: sodium isothiocyanate; HDAC: histone deacetylase; DDX: DEAD box family of RNA helicases; PP1: protein phosphatase one; PP2A: protein phosphatase 2A; LCP1: epidermal Langerhans cell protein; KPI-2: Kinase/Phosphatase/Inhibitor-2; NIPP: nuclear inhibitor of protein phosphatase-1: ZAP (ZAP3): YLPM-motif contain PP1 interactor; p99: PP1 interactor (also PNUTS); G_M_: skeletal muscle glycogen binding PP1 interactor; MC: microcystin; p84/Thoc1: THO complex 1; p54nrb/nono: 54 kDa nuclear RNA binding protein; NUP50: nucleoporin 50; TIP49a/RUVBL2: TBP-interacting protein 49/RuvB-like 2; PP4: protein phosphatase four; Repo-Man: recruits PP1 onto mitotic chromatin at anaphase; MBS85: myosin-binding subunit of 85 kDa; LRR-Ri: Leucine-rich repeats (LRRs): ribonuclease inhibitor; GRP78: Glucose-Regulated Protein of 78-kDa.

## Authors' contributions

GBGM, LTM and AIL conceived of the study, and participated in its design. GBGM, LTM, VD and MN carried out the biochemical studies and drafted the manuscript. DGC, RG and YWL carried out the mass spectrometry to identify proteins.

## Supplementary Material

Additional file 1 **Supplementary Figure 1. Identification of novel rat liver nuclear PP1 binding and complex proteins by displacement affinity chromatography**. Protein was extracted from isolated rat liver nuclei, incubated with microcystin-Sepharose, the matrix washed extensively and eluted with GKK**RVRW**ADLE peptide, followed by elution with 3 M NaSCN [31]. GKK**RVRW**ADLE and NaSCN eluted samples were concentrated separately to an equal volume and run on 10% SDS-PAGE and stained with Collodial blue. In a parallel experiment, the bands shown above were excised, trypsin digested and analyzed by mass spectrometry for identification. The top matched identified proteins for each band(s) are indicated to the left of the figure. Additional matches for each excised band and details of protein identifications are in Additional file 2 online. Click here for file

Additional file 2 **Additional file 2-Supplementary Table 1. Identification of proteins eluted from microcystin-Sepharose using an 'RVRW' peptide (Rattus norvegicus)**. Proteins were run on SDS-PAGE and stained with Collodial blue. Individual bands were excised, digested with trypsin and proteins identified by Mass Spectrometry. These proteins are shown by gel band excised with and protein name/description, gene accession numbers, gene name and whether they were previously identified has a PP1 targeting subunit (Known PP1 TS). Click here for file

Additional file 3 **Supplementary Figure 2. Identification of HeLa nuclear PP1 binding and complex proteins by displacement affinity chromatography**. The GKK**RVRW**ADLE elution from Figure 2a has been cropped and the top match identified proteins for each band(s) are indicated on the figure. Additional matches for each excised band and details of protein identifications are in Additional file 4 (Supplementary Table 2) online. Click here for file

Additional file 4 **Supplementary Table 2. Identification of proteins eluted from microcystin-Sepharose using an 'RVRW' peptide (Homo Sapiens)**. Proteins were run on SDS-PAGE and stained with Collodial blue. Individual bands were excised, digested with trypsin and proteins identified by Mass Spectrometry. These proteins are shown by gel band excised with gene accession numbers, gene name, number of peptide identified by mass spectrometry and whether they were previously identified has a PP1 targeting subunit (Known PP1 TS). Click here for file
