# Supplementary figures and images for: Displacement affinity chromatography of protein phosphatase one (PP1) complexes
Source: BMC Biochem. 2008 Nov 10;9:28. doi: 10.1186/1471-2091-9-28 (PMC2587467; doi:10.1186/1471-2091-9-28)

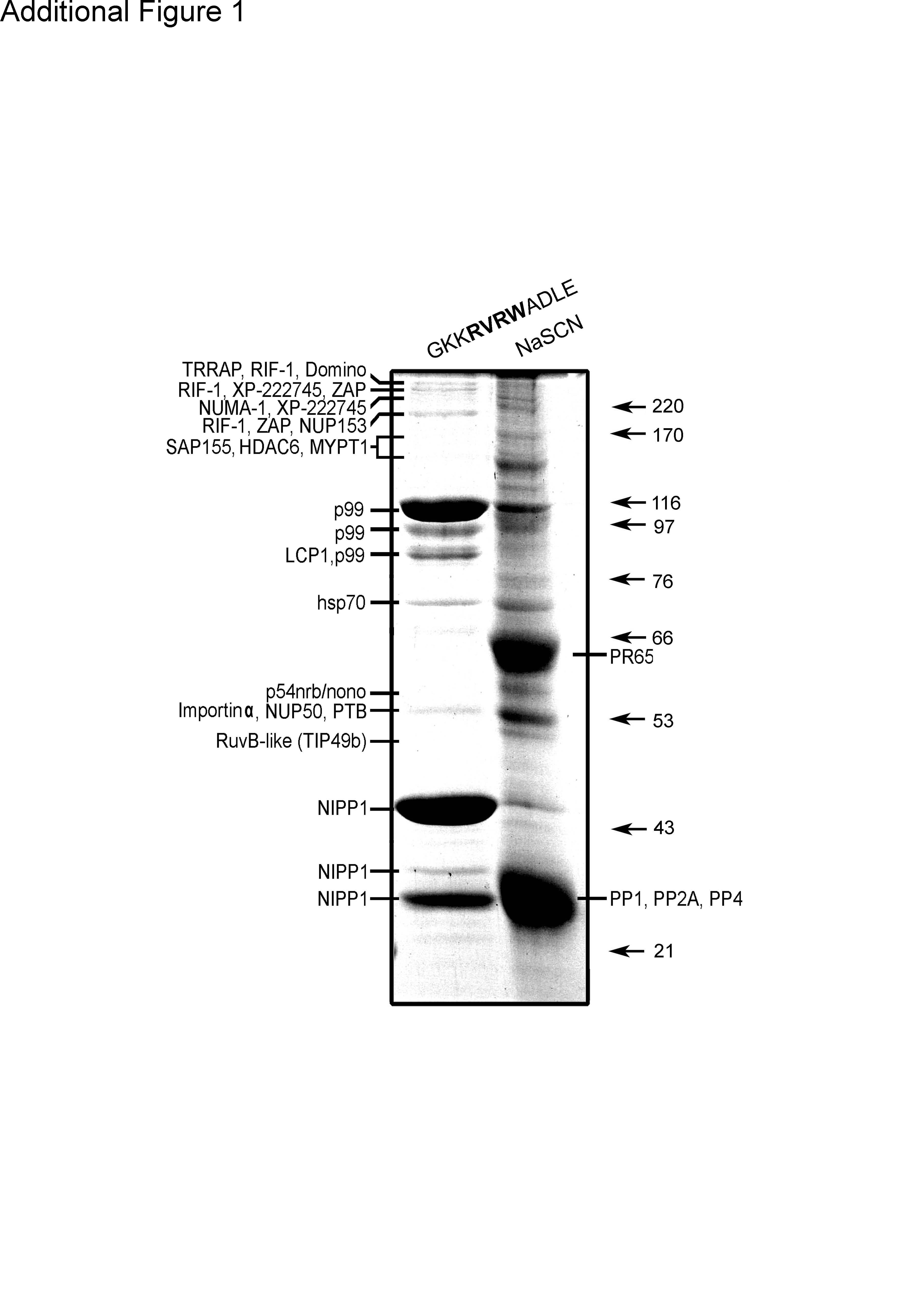

Supplement: Additional file 1 — Supplementary Figure 1. Identification of novel rat liver nuclear PP1 binding and complex proteins by displacement affinity chromatography. Protein was extracted from isolated rat liver nuclei, incubated with microcystin-Sepharose, the matrix washed extensively and eluted with GKKRVRWADLE peptide, followed by elution with 3 M NaSCN [31]. GKKRVRWADLE and NaSCN eluted samples were concentrated separately to an equal volume and run on 10% SDS-PAGE and stained with Collodial blue. In a parallel experiment, the bands shown above were excised, trypsin digested and analyzed by mass spectrometry for identification. The top matched identified proteins for each band(s) are indicated to the left of the figure. Additional matches for each excised band and details of protein identifications are in Additional file 2 online. [file 1471-2091-9-28-S1.jpeg]

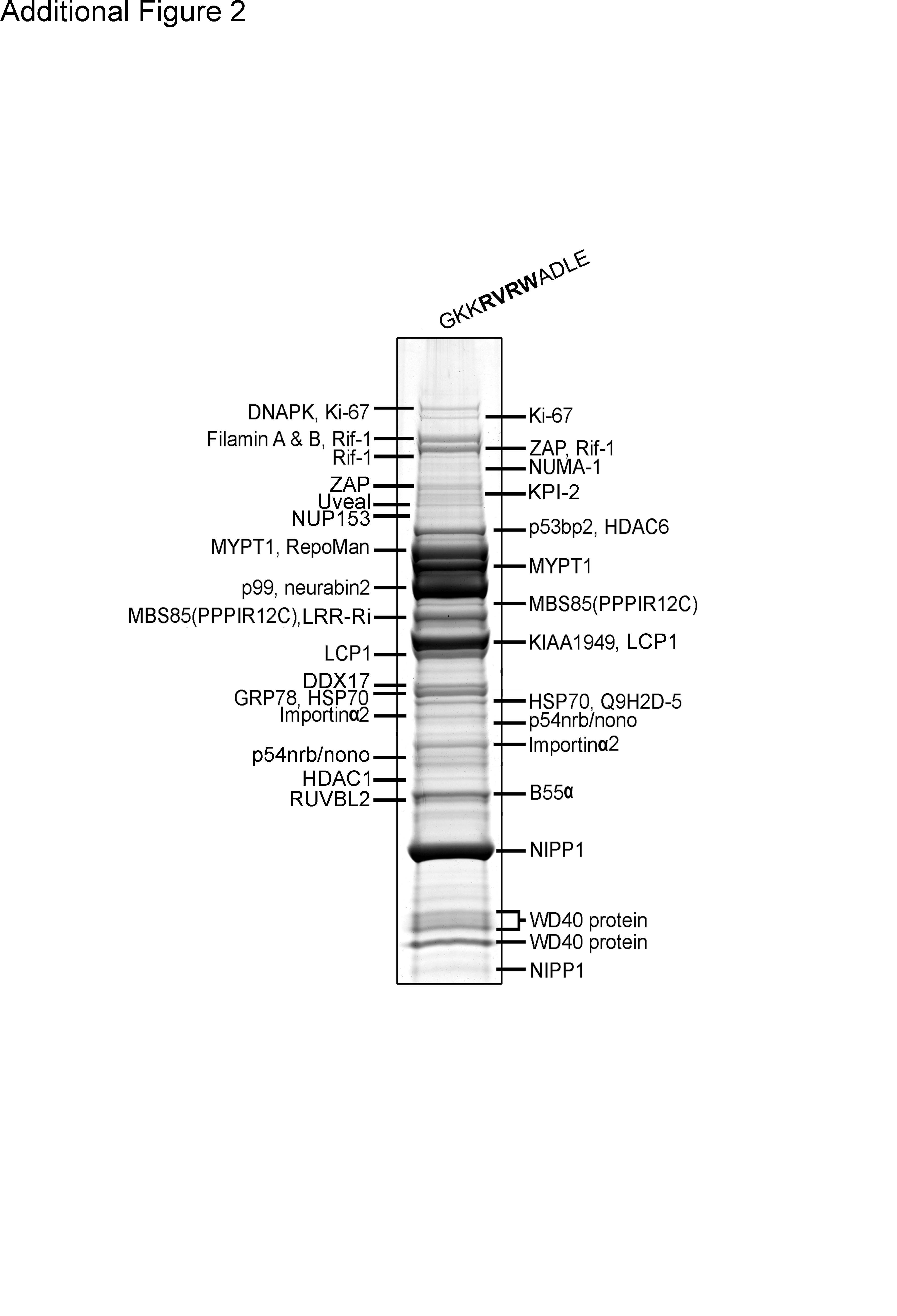

Supplement: Additional file 3 — Supplementary Figure 2. Identification of HeLa nuclear PP1 binding and complex proteins by displacement affinity chromatography. The GKKRVRWADLE elution from Figure 2a has been cropped and the top match identified proteins for each band(s) are indicated on the figure. Additional matches for each excised band and details of protein identifications are in Additional file 4 (Supplementary Table 2) online. [file 1471-2091-9-28-S3.jpeg]
